# Risk and Resilience in Trajectories of Post-Traumatic Stress Symptoms among First Responders after the 2011 Great East Japan Earthquake: a 7-year prospective cohort study

**DOI:** 10.1192/j.eurpsy.2022.629

**Published:** 2022-09-01

**Authors:** F. Van Der Does, T. Saito, M. Nagamine, N. Van Der Wee, J. Shigemura, T. Yamamoto, Y. Takahashi, M. Koga, H. Toda, A. Yoshino, E. Vermetten, E. Giltay

**Affiliations:** 1 Leiden University Medical Center, Psychiatry, Leiden, Netherlands; 2 School of Medicine, National Defense Medical College, Psychiatry, Tokorozawa, Japan; 3 National Defense Medical College Research Institute, Behavioral Science, Tokorozawa, Japan

**Keywords:** Trajectory Analysis, Post-traumatic stress disorder, First responders, Natural disaster

## Abstract

**Introduction:**

First responders to disasters are at risk of developing post-traumatic stress disorder (PTSD). The trajectories of post-traumatic stress symptom severity differ among individuals, even if they are exposed to similar events. These trajectories have not yet been reported in non-Western first responders.

**Objectives:**

We aimed to explore post-traumatic stress symptom severity trajectories and their risk factors in first responders to the 2011 Great East Japan Earthquake (GEJE)— a historically large earthquake that resulted in a tsunami and a nuclear disaster.

**Methods:**

56 388 Japan Ground Self-Defense Force (JGSDF) personnel dispatched to the GEJE were enrolled in this seven-year longitudinal cohort study. PTSD symptom severity was measured using the Impact of Event Scale-Revised (IES-R). Trajectories were identified using latent growth mixture models (LGMM). Nine potential risk factors for the symptom severity trajectories were analyzed using multinomial logistic regression.

**Results:**

Five symptom severity trajectories were identified: “resilient” (54.7%), “recovery” (24.5%), “incomplete recovery” (10.7%), “late-onset” (5.7%), and “chronic” (4.3%). The main risk factors for the four non-resilient trajectories were older age, personal disaster experiences, and working conditions. These working conditions included duties involving body recovery or radiation exposure risk, longer deployment length, later or no post-deployment leave, and longer post-deployment overtime.

**Conclusions:**

The majority of first responders to GEJE were resilient and developed few or no PTSD symptoms. A substantial minority experienced late-onset and chronic symptom severity trajectories. The identified risk factors can inform policies for prevention, early detection, and intervention in individuals at risk of developing symptomatic trajectories.

**Disclosure:**

No significant relationships.

